# Supplementary material for: Aqueous Heat Method for the Preparation of Hybrid Lipid–Polymer Structures: From Preformulation Studies to Protein Delivery
Source: Biomedicines. 2022 May 24;10(6):1228. doi: 10.3390/biomedicines10061228 (PMC9220230; doi:10.3390/biomedicines10061228)
Supplement: Supplementary file 1 [file biomedicines-10-01228-s001.zip › biomedicines-1732785-supplementary.pdf]

biomedicines-1709699

Aqueous heat method for the preparation of hybrid lipid-polymer structures: from preformulation studies to protein delivery

Natassa Pippa, Nefeli Lagopati, Aleksander Forys, Maria Chountoulesi, Hektor Katifelis, Varvara Chrysostomou, Barbara Trzebicka, Maria Gazouli, Costas Demetzos and Stergios Pispas

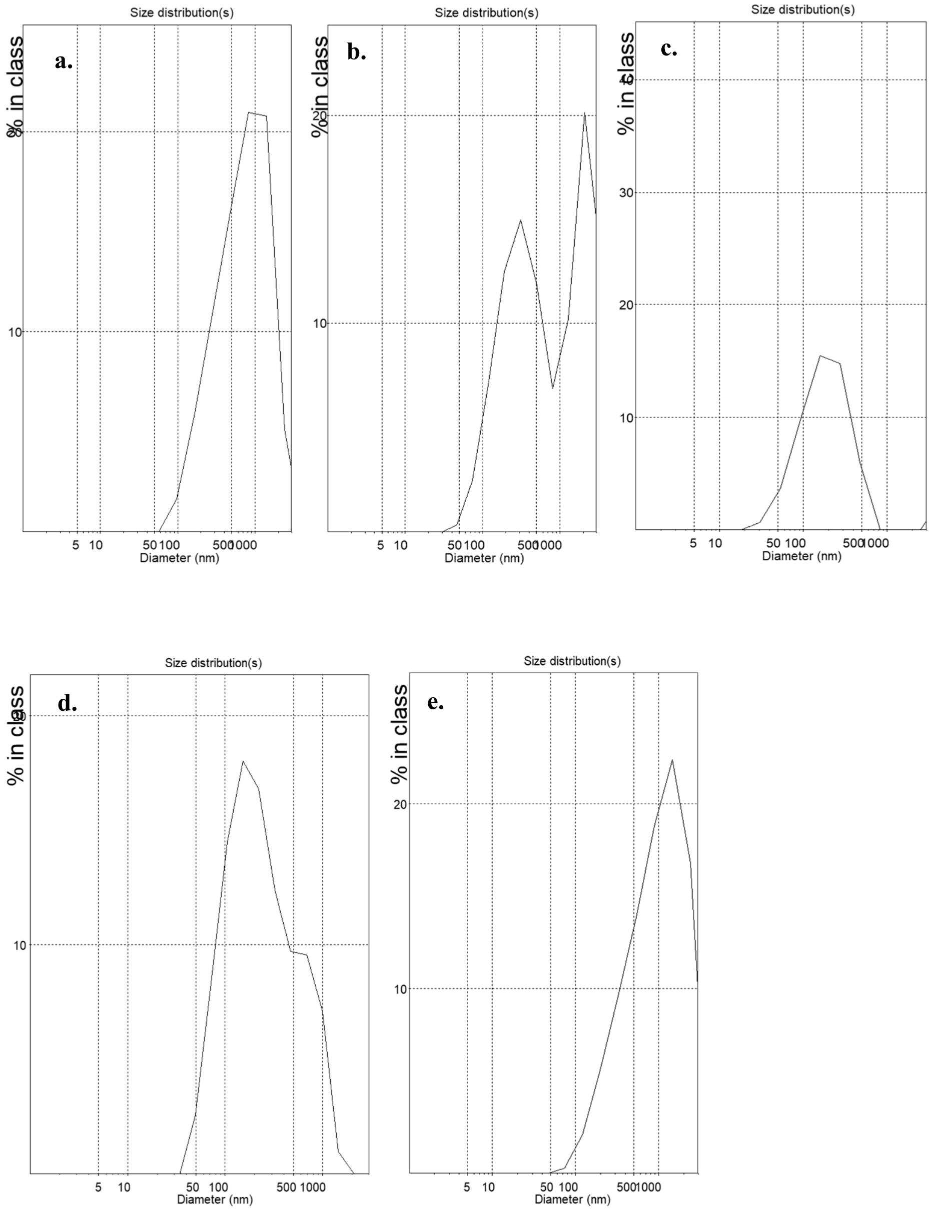

Figure S1. The size distributions of **a.** DDA:TDB 1:0.2 weight ratio; **b.** DDA:TDB:PLMA-b-PDMAEMA 1:0.2:0.5 weight ratio; **c.** DDA:TDB:PLMA-b-PDMAEMA 1:0.2:1 weight ratio; **d.** DDA:TDB:PLMA-b-PDMAEMA 1:0.2:2.5 weight ratio and **e.** DDA:TDB:PAMAM G4 1:0.2:0.1 weight ratio.

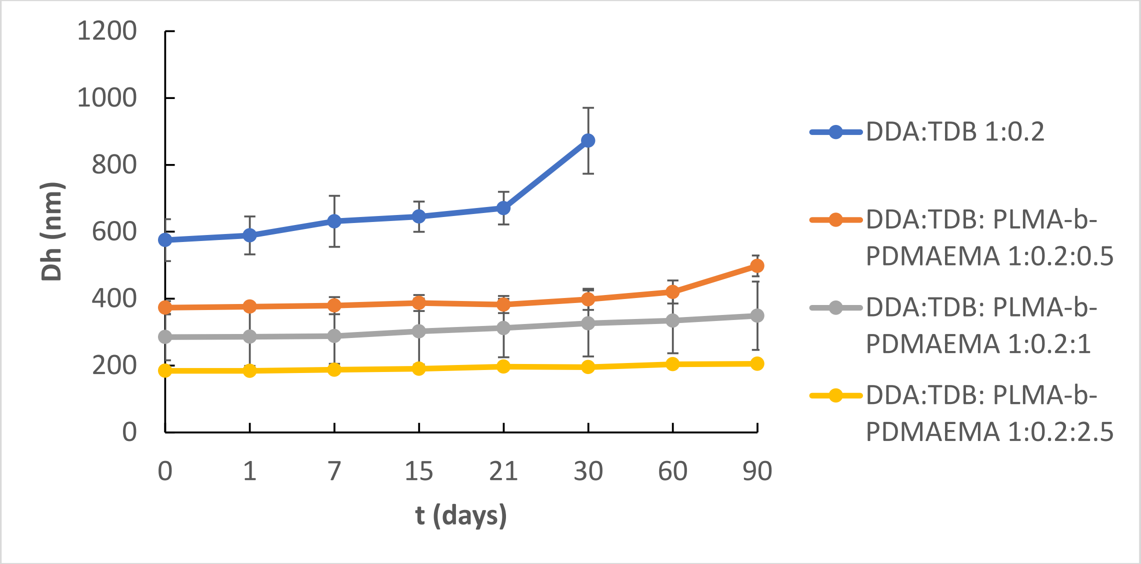

Figure S2. Stability assessment of the prepared systems.

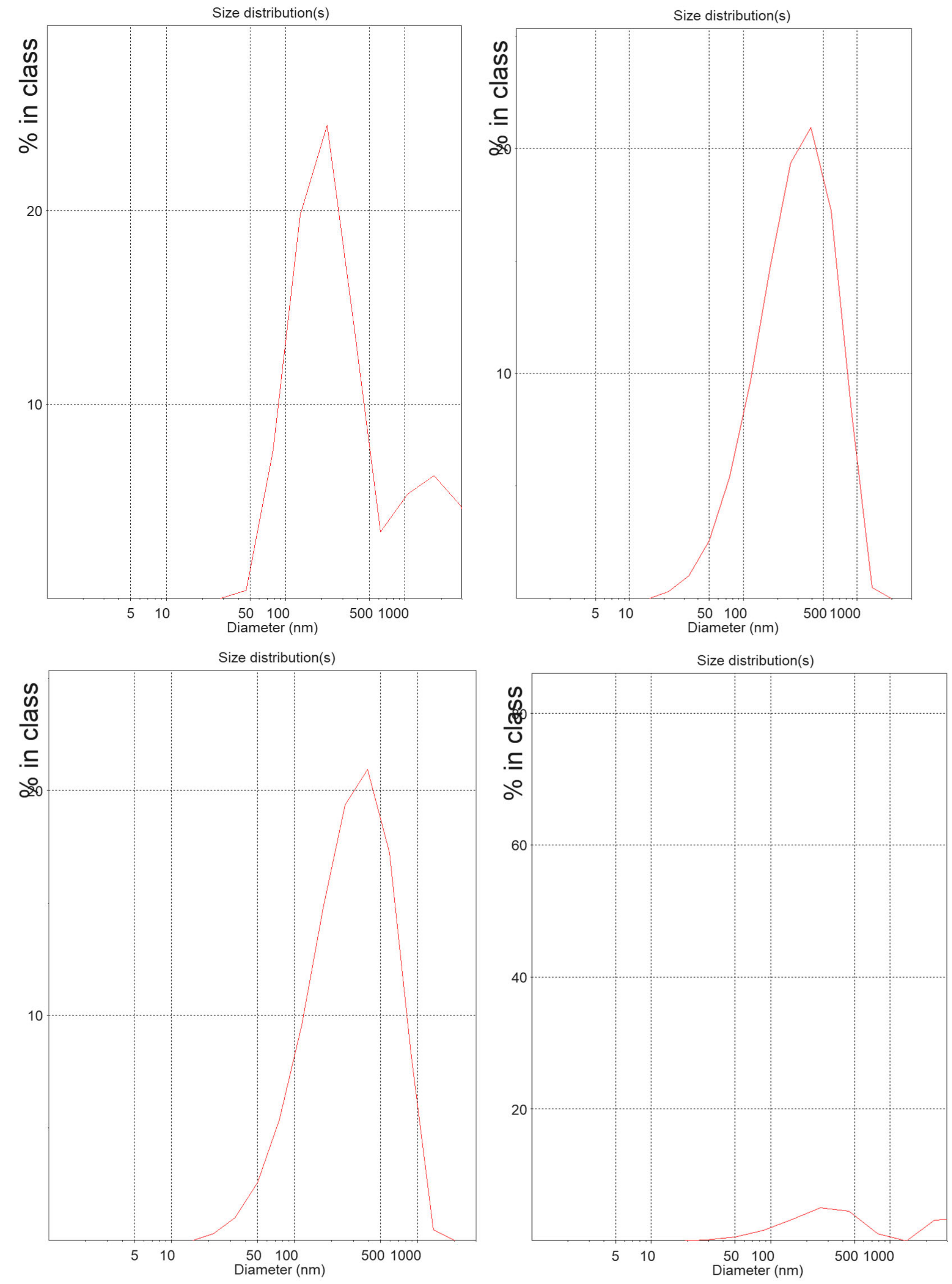

Figure S3. The size distributions of **a.** DDA:TDB:PLMA-b-PDMAEMA:BSA 1:0.2:0.5:1 weight ratio; **b.** DDA:TDB:PLMA-b-PDMAEMA:BSA 1:0.2:1:1 weight ratio; **c.** DDA:TDB:PLMA-b-PDMAEMA:BSA 1:0.2:2.5:1 weight ratio and **d.** DDA:TDB:PAMAM G4:BSA 1:0.2:0.1:1 weight ratio.
